# Editorial: The regulatory role of metabolic organ-secreted factors in the development of cardiovascular diseases

**DOI:** 10.3389/fcvm.2023.1246912

**Published:** 2023-07-13

**Authors:** Mei You, Cheng-Chao Ruan, Qiuhua Yang, James Philip Hobkirk, Peng Gao

**Affiliations:** ^1^ Department of Hypertension and Endocrinology, Center for Hypertension and Metabolic Diseases, Daping Hospital, Chongqing Institute of Hypertension, Army Medical University, Chongqing, China; ^2^ State Key Laboratory of Medical Neurobiology, Shanghai Key Laboratory of Bioactive Small Molecules, Department of Physiology and Pathophysiology, School of Basic Medical Sciences, Fudan University, Shanghai, China; ^3^ Vascular Biology Center, Medical College of Georgia, Augusta University, Augusta, GA, United States; ^4^ School of Life Sciences, University of Hull, Kingston upon Hull, United Kingdom

**Keywords:** cardiovascular disease, metabolic organ-secreted factor, nonalcoholic fatty liver disease, cardiac hypertrophy, myocardial infarction

**Editorial on the Research Topic** The regulatory role of metabolic organ-secreted factors in the development of cardiovascular diseases

Although metabolic disorders have been widely accepted to be a critical risk factor for cardiovascular diseases (CVDs), the mechanisms underlying the promotional effects of metabolic disorders on CVDs are not fully understood. As the central part of inter-organ communication, metabolic organs not only regulate blood glucose and blood lipid levels but also secrete many metabolic regulatory factors, such as fibroblast growth factor (FGF) 21, angiopoietin-like 4 (ANGPTL4), retinol-binding protein 4 (RBP4) secreted by the liver, adiponectin and leptin secreted by adipose tissue, myostatin and irisin secreted by muscle tissue, etc. These regulatory factors can regulate the structure and function of the heart and blood vessels, thus playing a vital role in the occurrence of CVDs (1). This Research Topic entitled “The Regulatory Role of Metabolic Organ-Secreted Factors in the Development of Cardiovascular Diseases” received three original articles and two review articles. This special issue focuses on recent findings to investigate the relationship between metabolic secretory factors and CVDs.

Metabolic syndrome (Mets) is related to a higher risk of cardiovascular outcomes and all-cause mortality (2). Emerging evidence has demonstrated that chronic short sleep may disturb the metabolism and result in adverse health outcomes, including hypertension, myocardial infarction (MI), stroke, coronary heart disease (CHD), diabetes mellitus, and impaired memory, which are regarded as risk factors for the progression of CVDs (3, 4). In this issue, Sun et al. found that short sleep duration is an independent risk factor for the development of CVDs, especially in people with Mets. In line with previous studies, this study suggests that the promotional effect of sleep deprivation on metabolic disorders should be related to low-level systemic inflammation induced by circadian rhythm disturbances. Similarly, our recent study has also determined that the liver would be the main source of low-level systemic inflammation that contributes to the development of salt-induced hypertension, as inhibiting hepatic steatosis and inflammation by metformin helped to relieve the elevated blood pressure and subsequent cardiovascular damage under high salt loading (5). These studies highlighted the metabolic organs as a potential therapeutic approach for controlling CVDs.

There is a growing number of epidemiological evidence that indicates nonalcoholic fatty liver disease (NAFLD) is strongly associated with an increased risk of major CVD events independently of traditional cardiovascular risk factors (6–8). One of the possible reasons may be that the liver serves as the most important dynamic metabolic organ and its secretory function disorder exerts powerful effects on metabolic processes both in the liver and in peripheral tissues (9). In this issue, Qin et al. provided a state-of-the-art review summarizing the inter-organ crosstalk between the liver and cardiovascular system, focusing on metabolic organ-secreted factors, including hepatokines, adipokines, cytokines, extracellular vesicles, and gut-derived factors, to emphasize that the underlying mechanisms accounting for the increased risk of CVDs in patients with NAFLD may be closely related to an abnormal expression and secretion of these factors, which result in glucose and lipid metabolic disorders, insulin resistance, oxidative stress, and chronic inflammation. Accordingly, Yang et al. summarized the effects of the major treatment of NAFLD on heart failure with preserved ejection fraction (HFpEF). This review listed a series of non-pharmacological treatment against NAFLD, such as dietary intervention and weight loss, and pharmacologic strategies currently applied in NAFLD for delaying the development of HFpEF, including statins, thiazolidinediones, glucagon-like peptide-1 (GLP-1) receptor agonists, sodium-glucose cotransporter 2 (SGLT2) inhibitors and metformin, thus providing a typical example for the treatment of CVDs by improving the function of metabolic organs. It is worth mentioning that the role of metabolic organ-secreted factors in CVDs is still being investigated and revealed. For example, our recent study has shown that a newly identified adipose-derived cytokine, asprosin, directly induces vascular endothelial-to-mesenchymal transition, which might contribute to the increased risk of peripheral vascular damage in type 2 diabetic patients (10). It can be predicted that these metabolic organ-secreted factors will become promising therapeutic targets for CVDs.

In addition to metabolic organs, many metabolic intermediates also play an active regulatory role in the development of CVDs (11), such as nicotinamide adenine dinucleotide (NAD) (12), lactate (13), and pyruvate (14). Wang et al. established a classic myocardial infarction (MI) model to evaluate the changes of myocardial and plasma Glycerophospholipid (GPL) profiles during the repair period after MI. They determined that the decrease in phosphatidylserine (PS) levels in the myocardium rather than plasma is an important contributor to MI injury, which is caused by the inhibition of phosphatidylserine synthetase 1 (PSS1). Moreover, PSS1 acts as a central regulator in myocardial damage and injury by reducing cardiomyocyte apoptosis, suggesting that targeting PSS1 in the heart may be an effective approach to attenuate MI injury. Therefore, different from metabolic factors involved in inter-organ crosstalk that would participate in chronic CVDs, changes in local metabolites of the cardiovascular system may be better indicators and therapeutic targets for acute cardiovascular injury.

Previous studies have demonstrated that abnormal expression or activity of regulatory molecules in the process of lipid production and utilization also contributes to the development of CVDs. As a crucial regulator of metabolic homeostasis, peroxisome proliferator activated receptor γ (PPARγ) not only plays an important physiological role in glucose metabolism, adipocyte differentiation, lipid storage, but also maintains vascular homeostasis (15, 16). However, the side effects of PPARγ agonists, sodium and fluid retention, hampered their clinical application. Wang et al. identified a new anti-hypertrophic PPARγ stabilizer, luteolin, by using a luciferase reporter-based high-throughput screening. In the transverse aortic constriction (TAC) model, luteolin effectively ameliorated pathological cardiac hypertrophy, fibrosis, metabolic disorder, and heart failure. Consistently, luteolin dose-dependently blocked phenylephrine-induced cardiomyocyte hypertrophy and improved myocardial fatty acid and glucose metabolism in cardiomyocyte. Furthermore, they verified luteolin direct binds to PPARγ, suppressing its ubiquitination and subsequent proteasomal degradation. This study indicates that the strategies of stabilizing PPARγ is a promising way for pathological cardiac hypertrophy and HF treatment, which may avoid the side effects of previous PPARγ agonists.

In conclusion, the articles published on this Research Topic have illustrated the role of metabolic syndrome, metabolic organs, and metabolites in the development of CVDs and have discovered a new drug beneficial to CVDs by improving metabolism. Further studies on this topic are important, and advances in this field can significantly improve our understanding of CVDs and would provide novel and alternative therapeutic strategies.
